# Design and Use of Nanostructured Single-Site Heterogeneous Catalysts for the Selective Transformation of Fine Chemicals

**DOI:** 10.3390/molecules15063829

**Published:** 2010-05-26

**Authors:** Vladimiro Dal Santo, Francesca Liguori, Claudio Pirovano, Matteo Guidotti

**Affiliations:** 1 CNR-Istituto di Scienze e Tecnologie Molecolari, Via C. Golgi 19, 20133, Milano, Italy;E-Mails: v.dalsanto@istm.cnr.it (V.D.S.); claudio.pirovano@unimi.it (C.P.); 2 CNR-Istituto di Chimica dei Composti OrganoMetallici, via Madonna del Piano, Sesto F.no (FI), Italy; E-Mail: francesca.liguori@iccom.cnr.it (F.L.)

**Keywords:** heterogeneous catalysis, single sites, fine chemistry, nanostructured oxides, selectivity

## Abstract

Nanostructured single-site heterogeneous catalysts possess the advantages of classical solid catalysts, in terms of easy recovery and recycling, together with a defined tailored chemical and steric environment around the catalytically active metal site. The use of inorganic oxide supports with selected shape and porosity at a nanometric level may have a relevant impact on the regio- and stereochemistry of the catalytic reaction. Analogously, by choosing the optimal preparation techniques to obtain spatially isolated and well-characterised active sites, it is possible to achieve performances that are comparable to (or, in the most favourable cases, better than) those obtained with homogeneous systems. Such catalysts are therefore particularly suitable for the transformation of highly-functionalised fine chemicals and some relevant examples where high chemo-, regio- and stereoselectivity are crucial will be described.

## 1. Introduction

Catalysis is principally divided in two branches: *homogeneous catalysis*, when the catalyst is in the same phase as the reaction mixture (typically in liquid phase), and *heterogeneous catalysis*, when the catalyst is in a different phase (typically solid/liquid, solid/gas or solid/liquid/gas). Table 1 summarizes a comparison between the main features of homogeneous and heterogeneous catalysis.

**Table 1:** Schematic comparison between homogeneous and heterogeneous catalysis.

Feature	Homogeneous Catalyst	Heterogeneous Catalyst
Form	*metal complex*	*solid, often metal or metal oxide*
Activity	*high*	*variable*
Selectivity	*high*	*variable*
Reaction Conditions	*mild*	*drastic*
Average Time of Life	*variable*	*long*
Sensitivity to Poisons	*low*	*high*
Problems of Diffusion	*none*	*possible*
Recycling	*difficult (and expensive)*	*easy*
Separation from Products	*difficult*	*easy*
Variations of steric and electronic features	*possible*	*difficult*
Intelligibility of the mechanism	*possible*	*difficult*

One of the main advantages of homogeneous molecular catalysts, when they operate under ideal conditions, is that their active sites are spatially well separated from one another, just as they are in enzymes. Because of such spatial separation and the self-similarity of the structures of the sites, there is a constant energetic interaction between each active site and the reactant (substrate). Another advantage, also a direct consequence of spatial isolation and energetic constancy, is that such catalysts are readily amenable to almost all the techniques of characterization as time-resolved NMR, FT-IR, X-Ray absorption and all other spectroscopic (and calorimetric) methods available. Nevertheless, although homogeneous catalysts present, in general, high activity and selectivity values under mild reaction conditions, they show, as a major drawback, a difficult recycling and separation from the products.

Heterogeneous catalysis has a fundamental role in the development of sustainable industrial processes, as it potentially possesses the ability to achieve the objectives of industrial catalysis, while paying attention to the principles of sustainable and green chemistry [1]. Easy separation, easy recovery, no problems in solubility and miscibility are the strengths of a heterogeneous system in order to reduce the cost of production and to set up environmentally benign processes. However, further efforts are necessary in the design of new heterogeneous catalysts to reach activity and selectivity values competitive with homogeneous ones.

Understanding structure/reactivity/selectivity relationships in classical metal-based heterogeneous systems is complicated by uncertainties in the active site structure(s) and in the large variety of surface species which are catalytically significant. In general, solid catalysts working heterogeneously possess a broad spectrum of active sites, each with their own energetics, activity and selectivity. For instance, in a dispersed metal particle, atoms located at surface steps, kinks or terraces or at flat exterior surface are stereochemically different from one another and this has a dramatic influence on the variety of energetic situations associated with the adsorption of substrate species [2,3]. Such complex situation is encountered not only with all metal- and alloy-based catalysts, but also with a very large number of other catalysts that are continuous solids, including close-packed oxides, halides and chalcogenides.

## 2. Single-Site Heterogeneous Catalyst

The past two decades have witnessed a remarkable progress in transferring the homogeneous molecular approach to heterogeneous catalysis, leading to the development of *single-site* heterogeneous catalysis [4,5,6,7,8,9]. A single-site heterogeneous catalyst (SSHC) is a solid where the catalytically active sites are well-defined, evenly distributed entities (*single sites*) with a definite chemical surroundings, as in conventional homogeneous systems, but which show all the advantages of heterogeneous systems, in terms of easy separation, recover and recyclability. Such single sites are typically located over solid supports with high surface area and present the following general features:

- they consist of one or few atoms (as in the case of chemically defined metal clusters);
- are spatially isolated from one another;
- have all identical energy of interaction between the site itself and a reactant;
- are structurally well characterized.

SSHC can be juxtaposed to those solid catalysts where the active species are present as metal particles or bulk domains of a metal or oxidic component whose composition is different from that of the support. In classical heterogeneous catalysts the catalytically active sites can be surrounded by atoms (or species) of the same nature (e.g., the Ni atoms in a conventional Ni/Al_2_O_3_ hydrogenation catalyst), whereas the atomic (or molecular) isolation of the active moieties is the distinctive feature of SSHC.

### 2.1. Preparation of single-site heterogeneous catalysts on inorganic supports

The host matrixes where single-site centres can be uniformly dispersed have inorganic, organic or composite nature. Inorganic matrices are typically oxidic solids (silicates, aluminosilicates, aluminophosphates, *etc*.), often with a high porosity and a very large specific surface area (in the order of 100 to 1,000 m^2^ g^-1^), such as crystallographically ordered microporous molecular sieves (zeolites and zeotypes) or non-ordered mesoporous materials. Organic host matrices have, on the contrary, a carbon-based backbone and are constituted of polymers with different side functionalities that are able to bind and accommodate the active single site. Then, composite matrices are composed by mixtures of inorganic and organic constituents, in variable proportions and with various morphologies (e.g., co-polymers, multiple-shell materials, mesoporous hybrid frameworks). In the present brief overview, the focus will be limited to inorganic matrixes only.

The first examples of SSHC can be found in the pioneering works by some authors, such as Ballard *et al*. or Candlin and Thomas, in the 1970s, when single-site silica supported systems began to show interesting results as catalysts, especially in polymerization and disproportionation of olefins [10,11,12,13]. Then, in the 1980s, some open-structure zeolitic aluminosilicates or open-structure aluminophosphates (AlPOs), where isolated heteroatoms (other than Si, Al and P) are dispersed in the framework of the zeolite or zeotype, appeared and they can be also considered early examples of SSHC, as they fulfil the main requirements of isolation, even distribution and definition of the chemical environment of the active sites [14,15,16,17,18,19]. Redox-active zeolites, such as titanosilicalite-1, or M-AlPOs (M = Mg, Co, Mn, Zn, *etc*.) belong to this class of catalysts. Finally, in the 1990s, thanks to the development of silica-based molecular sieves in the mesopore range, larger substrate molecules can enter the pore network, be processed there and leave it [20,21,22]. Some well known mesoporous materials, that are ideal supports for SSHC, are, for instance, MCM-41 [23], MCM-48 [24], HMS, MSU [25,26], KIT-6 [27], SBA-15 or SBA-16 [28]. These materials, when compared to microporous crystalline zeolites, exhibit higher surface area (600-1,300 m^2^ g^-1^), and larger pore size (2–30 nm) and may, in principle, possess various potential applications in industrial catalytic reactions [29]. Indeed, the recent start up of production plants at large-scale level (up to 20 t yr^-1^) for the production of nanostructured mesoporous silicas make these materials now suitable for a number of different applications in commodity and fine chemicals synthesis [30,31].

**Figure 1:**
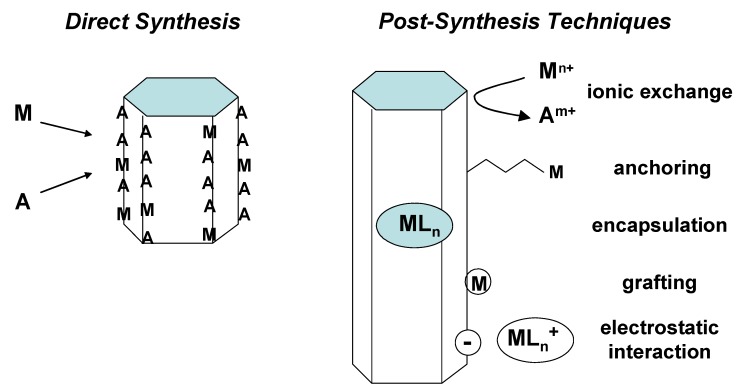
Functionalization of inorganic oxide supports. Several strategies and pathways are possible to introduce novel functions into or onto molecular sieves. M: catalytically active metal (e.g., Ti); A: non-active (e.g., Si); L: ligand.

More generally, single sites can be added to a pre-existing support by *post-synthesis* techniques: the isolated sites can be deposited, heterogenised, tethered or linked to a previous matrix. Otherwise, the catalytically active single sites can also have a structural role, when they are inserted in the framework or in the lattice of a spatially ordered material, via *direct synthesis* (Figure 1). This is the case, for instance, of metal-containing zeolites, where Si or Al atoms are isomorphically substituted by redox metal centres, or of the heteroatoms of an oxidic nanoporous solid.

SSHC can display a *redox*, an *acid* or a *basic* catalytic activity according to the nature of the site, as well as a *bifunctional* character when two (or more) active centres are present. Actually, two different (sometimes incompatible) functionalities can often coexist on the same solid (such as acid and redox, basic and redox or, even, acid and basic), thanks to the spatial segregation of the sites which avoids their mutual annihilation [32,33]. This last feature is peculiar of heterogeneous solid catalysts and it cannot be found in homogenous catalysis

With regard to the methods of creation of single-site centres, we can roughly list three main approaches to obtain single sites from their precursors:

1) in-matrix synthesis,
2) post-synthetic covalent deposition,
3) post-synthetic non-covalent deposition.

In the first case, the atom-isolated catalytically active sites are homogeneously dispersed in the matrix of the support and they are located at or adjacent to ions that have replaced framework ions of the parent structure. The precursor of the active species is already present in the synthesis mixture of the final material, together with the other components, and the single sites are introduced during the synthesis step (e.g., during the hydrothermal synthesis of a zeolite or the co-precipitation step of an amorphous mixed oxide). The chemical environment of the site (in terms of hydrophilic/hydrophobic character, surface acidity, steric constraints, *etc*.) is strongly dependent on physical-chemical characteristics of the matrix. As a main drawback, some centres can be ‘buried’ within the bulk of the solid and they can be not accessible and available for catalysis. Moreover, the presence of ‘heteroatoms’ in the synthesis mixture may affect critically some preparation phases (gelation, crystallization, condensation, *etc*.) and it is then often necessary to set up a completely new synthesis protocol for every new desired material. Such approach can be very time-consuming, but the possibility of regeneration of these catalysts is generally better than the one of catalysts obtained via post-synthesis techniques. In fact, they can easily withstand calcination and severe washing treatments without losing their structural stability and chemical integrity. In addition, thanks to a careful control during the preparation of highly mesostructured functionalized materials containing homogeneously distributed organic moieties along their channels, it is possible to obtain a regular distribution of the organic fragments containing the catalytically-active metal single sites in the final material [34].

**Figure 2:**
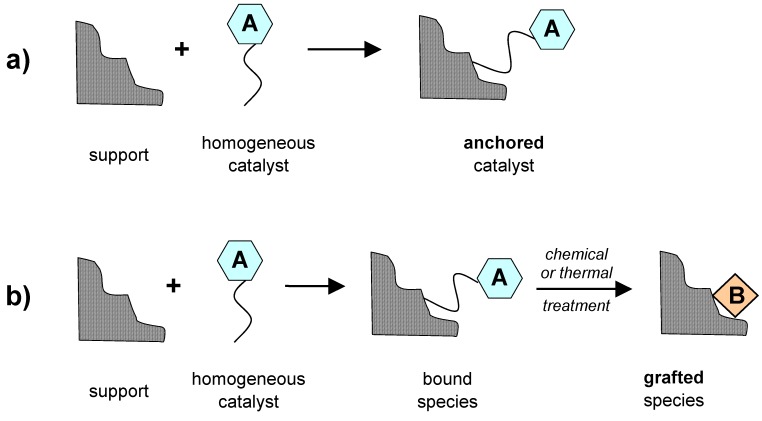
Post-synthesis covalent functionalization of inorganic oxide supports: anchoring and grafting.

In the second case, by means of post-synthesis covalent deposition, it is possible to obtain relatively easily single-site catalysts from well-known solids (some of them can also be commercially available). The active centre is generally added to the support as a precursor that can be deposited irreversibly (more precisely, *anchored* or *grafted*) onto the surface as it is, by the formation of covalent bonds, or after a functionalisation with a side chain (a tether) [35,36]. In the *anchoring* technique, the active metal (the single site) maintains the definite chemical surroundings as in the parent homogeneous precursor (the covalent deposition takes place only at the opposite end of the tether) with all the advantages of heterogeneous systems, namely easy separation, recover and recyclability (Figure 2a). Conversely, in the *grafting* technique, the active single site has a different chemical surrounding with respect to the parent precursor, since the coordination shell around the metal centre is partially modified during the covalent deposition, and a new reactivity of the active species can be expected (Figure 2b).

**Figure 3:**
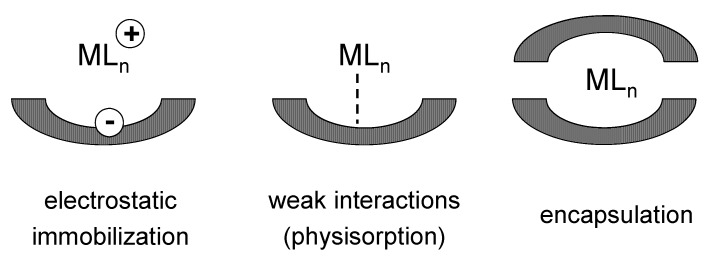
Post-synthesis non-covalent deposition of metal complexes onto inorganic oxide supports.

Finally, in the third case, the homogeneous precursors of the single-site active centres are immobilized onto the surface of solid supports by non-covalent interactions, such as hydrogen bonds, weak van der Waals interactions or by mechanical confinement (trapping or encapsulation) (Figure 3). In particular, encapsulation covers a large selection of methods for immobilising catalytically active species within the pores of inorganic or organic solids and it allows one to keep unaffected the optimal performances of the original homogeneous catalysts. In some cases, thanks to a positive cooperative effect, it is also possible to have a final system with improved characteristics with respect to the parent precursor. For instance, encapsulated metal complexes are assembled *in situ* by intrazeolite synthesis and complexation and, for this reason, they are often referred to as ‘*ship-in-the-bottle* complexes’ [37,38,39]. Then, once formed, the metal complexes are spatially entrapped in the molecular sieve, since they are too bulky to diffuse out, whereas the reactants and the products can freely move through the pores. This kind of materials often show, with respect to the homogeneous parent complex, enhanced: (1) activity, thanks to zeolite sorption and/or concentration effects; (2) selectivity, as detrimental free-radical side reaction, that occur in solution, are mostly suppressed in encapsulated systems, and (3) stability, since catalyst deactivation pathways are hindered by local site isolation of the complexes inside the solid matrix. Encapsulated metal complexes can also easily be separated from the reaction media and reused, with no metal leaching.

Many approaches have been created and are being developed for preparing SSHC, as it is possible to use different support materials and a wide range of metal precursors of different nature. Furthermore, there are many possibilities to control and modify the immediate atomic environment and the central atomic structure of the active site, in order to tune the features of the catalyst according to its applications.

## 3. Selected Examples and Applications of SSHC

The panorama of single-site heterogeneous catalysts applied to the selective transformation of richly functionalised chemicals (such as fine and specialty chemicals) is wide and multifaceted. Here we report some examples developed in the field, selected according to the method of preparation of the SSHC and to the most relevant catalytic applications displayed so far.

### 3.1. Titanosilicalite-1 (TS-1): the forerunner of in-matrix single-site catalysts

**Figure 4:**
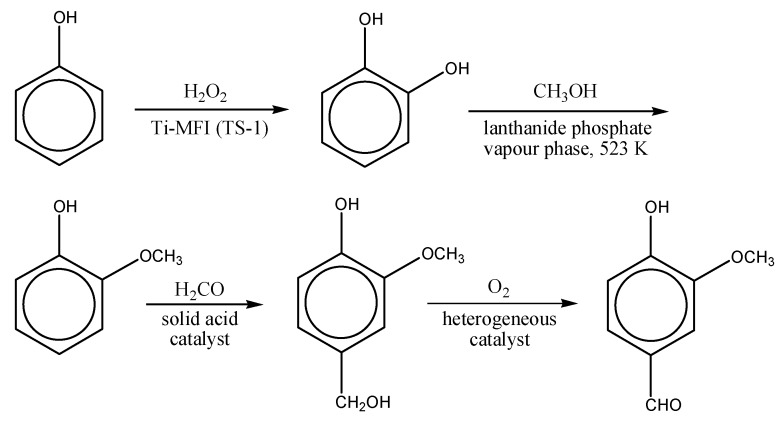
From phenol to vanillin, according the process developed by Rhodia.

The discovery of titanosilicalite-1 (TS-1) at the beginning of the 1980s constituted a major breakthrough in oxidation catalysis and can be considered the forerunner of redox-active single-site catalysts prepared by direct in-matrix synthesis [40,41]. Titanium(IV) centres are evenly distributed within the aluminosilicate crystalline framework of a zeolite with MFI topology (analogous to ZSM-5 zeolite). The outstanding efficiency and activity of this zeolite in selective oxidations with diluted aqueous hydrogen peroxide are commonly attributed to two main factors [42]: 1) the isolation of titanium sites, preventing the undesired decomposition of H_2_O_2_, which is induced and catalyzed by clusters of adjacent titanium sites (such as anatase TiO_2_ domains) and 2) the hydrophobic character of the lattice, enabling the preferential adsorption of the hydrophobic substrates in the zeolite micropores in the presence of water. Up to now, TS-1 has shown well-established efficiency in series of oxidation processes with hydrogen peroxide: namely, epoxidation of alkenes, oxidation of alkanes, oxidation of alcohols, cyclohexanone ammoximation and phenol hydroxylation [43,44]. In all these processes, TS-1 proved to be very active (in most reaction the conversion of the substrate is almost complete) and extremely selective (for instance, with selectivity values up to 94% in phenol hydroxylation to hydroquinone and pyrocatechol [45] or up to 98% in EniChem-Sumitomo cyclohexanone ammoximation process [46]). In the field of fine chemicals manufacture, TS-1 has found a remarkable application in the process for the manufacture of vanillin, a major flavour ingredient, from phenol, developed by Rhodia. In the whole process, one equivalent of phenol, hydrogen peroxide, methanol, formaldehyde and molecular oxygen are converted into one equivalent of vanillin and three equivalents of water (Figure 4) [47]. Site isolation and definition is achieved in TS-1 thanks to the high dispersion of Ti sites throughout the zeolite lattice (Ti content in titanosilicalites is around 1–2 wt.%) and to the controlled hydrothermal synthesis conditions. The main drawback of the microporous titanium silicates is the small size of the pores, which prevents their use for the conversion of large organic molecules. In particular, the system TS-1 + H_2_O_2_works only with substrates with a kinetic diameter smaller than 0.6 nm.

The successful application of TS-1 in various relevant oxidation reactions prompted the scientific community to investigate how to insert other redox-active metals into the lattice of zeolites with various topologies. After several and repeated attempts, many metal-containing zeolites did not prove to be as reliable as TS-1 and, in numerous examples, the new solids showed a poor stability towards metal leaching (e.g., chromosilicalite-1, CrS-1, and vanadosilicalite-1, VS-1, release non-negligible amounts of metal into the liquid phase under reaction conditions) or reduced structural robustness [48]. So far, only few redox-active microporous molecular sieves have proven to be truly heterogeneous, stable and easily recyclable. Among them, it is worth mentioning: 1) Sn(IV)-substituted or Zr(IV)-substituted beta zeolite (Sn-BEA; Zr-BEA) [49,50] and 2) the large family of metal-containing aluminophosphates (AlPOs) [51]. Both of them showed a real potential for a large-scale industrial application.

**Figure 5:**
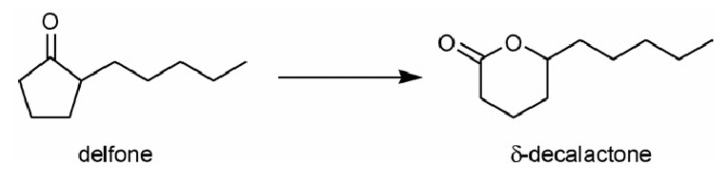
Baeyer-Villiger oxidation of delfone into δ-decalactone over Sn-BEA zeolite.

In particular, in Sn-BEA, isolated tetrahedral tin sites are inserted and homogeneously dispersed into the BEA zeolite framework and they showed noteworthy and peculiar activity for the Baeyer-Villiger oxidation of cyclic ketones, aromatic aldehydes and α,β-unsaturated aldehydes. Detailed mechanistic studies confirmed that the catalytically active site is due to the occurrence of partially hydrolysed framework Sn centres, where a Lewis acid site, located on Sn(IV), and an adjacent basic site, on the oxygen atom of the Sn-OH group formed upon hydrolysis, are simultaneously present. Sn-BEA plus hydrogen peroxide gives therefore an alternative to the use of conventional oxidants, such as peroxoacids, in Baeyer-Villiger oxidations [52]. Sn-BEA was also tested in the oxidation of delfone into δ-decalactone (an important creamy and fruity aroma for the flavours and fragrances industry; Figure 5) in a stirred batch reactor. The desired product was obtained in 86% yield, even with low amounts of catalyst (*ca*. 0.5 wt.%) and turn-over numbers as high as *ca*. 10,000 were achieved, confirming the high stability of the catalytic system [51].

Similarly, Al-free Zr-BEA proved to catalyse the Meerwein-Ponndorf-Verley (MPV) reduction of α,β-unsaturated aldehydes to the corresponding alcohols with high selectivity and good stability. It showed good activity in the cyclisation of citronellal to isopulegols with >97% chemoselectivity and, remarkably, a high diastereoselectivity for (±)-isopulegol of *ca*. 93% [53]. In the MPV reduction of cinnamaldehyde, a high initial TOF of 50–80 mol mol^-1^_Zr_ h^-1^ was obtained over Al-free Zr-BEA samples, the selectivity to cinnamyl alcohol being >98% throughout the reaction. In contrast, Al-containing Zr-BEA samples were less active and selective, as the presence of Brønsted acid sites in the Al-containing samples catalysed the formation of 1-cinnamyl 2-propyl ether from cinnamyl alcohol and 2-propanol [54].

The second class of stable and reliable redox-active molecular sieves is the family of microporous aluminophosphate (AlPOs), zeotype crystalline materials where isolated redox-active sites and acid centres coexist and are accommodated by utilizing low degrees of isomorphous substitutions (only 4% of the total Al atoms). For instance, catalysts of the general formula M(II)M(III)-AlPO-36 are structurally defined molecular sieves with pore openings of 0.65 × 0.75 nm and a surface area of *ca*. 700 m^2^ g^-1^ and are able to catalyze, in one apparent step, in liquid-phase under solvent-free conditions, the synthesis of ε-caprolactam from cyclohexanone, ammonia and molecular oxygen from air. In these systems, M(II) ions have a proton loosely bound to an adjacent framework oxygen atom and thus show a Brønsted acid behaviour, whereas M(III) ions are redox-active sites capable of activating molecules such as hydrocarbons and oxygen [55]. In best cases, the reaction takes place at 80 °C under 35 bar of air in pure cyclohexanone and, over Mg(II)_0.02_Mn(III)_0.02_-Al_0.96_PO-36, selectivities to ε-caprolactam as high as 78% were obtained at 68% conversion of cyclohexanone [56]. Similarly, Mn(III)-substituted AlPOs with AFI morphology (Mn-AlPO-5) were applied together with acetylperoxyborate, as a source of active oxygen, to the selective oxidation of picolines, pyrazines and pyridazines to the related carboxylic acids, with a limited production of *N*-oxides, as by-products. In particular, 4-picoline was converted into isonicotinic acid in good yields (up to 70%) and remarkable selectivities (higher than 90%) in the absence of organic solvents [57].

### 3.2. Grafted single-site transition metals supported onto high surface area silica

**Figure 6:**
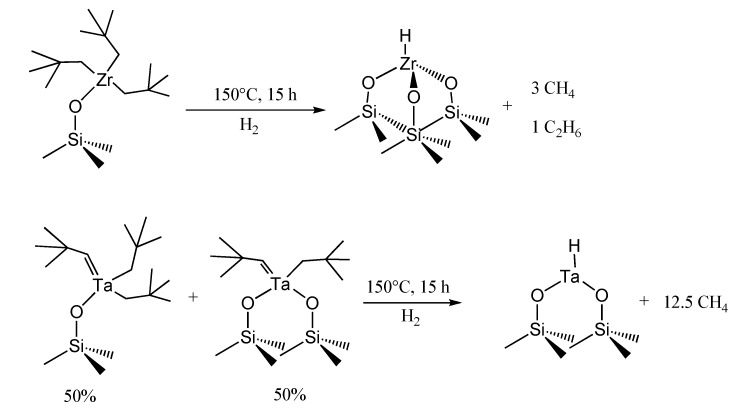
Grafted single-site Zr hydride and Ta hydride complexes as heterogeneous catalysts for the activation of light alkanes.

Twenty-five years ago Basset and co-workers introduced the concept of *surface organometallic chemistry* [58], starting a work based on the systematic study of the reactivity of organometallic complexes or coordination compounds with the surface of support materials, in particular functionalizing the surface of a non-porous pyrogenic (Aerosil) silica [4,59], and developing the idea according to which a metal oxide behaves as a ligand for a surface organometallic molecular species [60,61]. Their work produced SSHC before the advent of ordered mesoporous silicas, by appropriately functionalizing the residual pendant silanol groups on Aerosil (whose typical surface area is 200 m^2^g^-1^ and silanol surface concentration 0.7 ± 0.2 per nm^2^, equivalent to 0.23 mol(OH) g^-1^). Specific examples of grafted single-site transition metals using surface organometallic chemistry are silica-supported tantalum hydride and silica-supported zirconium hydride, respectively a bipodally-grafted and a tripodally-grafted system. These compounds are catalysts for the activation of small alkanes under mild conditions. With Zr hydride complex the hydrogenolysis of neopentyl ligands produced a 3:1 mixture of methane and ethane [62], while, with Ta hydride and in the presence of H_2_, alkanes were transformed into methane as the sole product [63] (Figure 6).

Later, the preparation of a large variety of SSHC began once it became readily possible to prepare mesoporous silica as inorganic supports (*vide supra*). Their inner and outer surfaces have a profusion of pendant silanol groups (approximately 1–2 per nm^2^) and they are optimal loci for covalently immobilizing transition metal ions with a specific catalytic (mostly redox) function. 

In this scenario, the development of solid catalysts for the selective oxidation of fine chemicals can be taken as an exemplary case. Since TS-1 is active only in the oxidation of small substrates, finding a SSHC suitable in the oxidation of bulky and richly functionalised molecules was (and still is) a task of major importance. Corma [64], Thomas [65,66], Pinnavaia [25] and others were quick to appreciate the advantages that large-pore silicas would confer in facilitating the preparation of single-site, metal-centered heterogeneous catalysts, aiming at obtaining solids with a good oxidation ability (as good as TS-1), but with high accessibility (as high as mesoporous silicas). A significant advance was made when Ti(IV) active centres were grafted onto the inner walls of MCM-41 mesoporous silica using an organometallic precursor, in particular titanocene dichloride [Ti(Cp)_2_Cl_2_] (Cp = C_5_H_5_), by Maschmeyer *et al*. [67]. This sort of Ti(IV)-containing mesoporous silica proved to be active and selective in the epoxidation of cycloalkenes with high selectivity (always higher than 90%) and good activity (grafted catalysts showed a specific activity *ca.* 10 times higher than the in-matrix Ti-MCM-41 materials). Later, by optimizing the experimental conditions, titanocene-grafted silicas showed interesting results in the epoxidation of a wide variety of unsaturated substrates: alcoholic terpenes (e.g., carveol epoxide was obtained with 82% conversion and 73% selectivity in 24 h) or unsaturated fatty acid methyl esters (e.g., castor oil methyl ester is epoxidised in 24 h with 97% conversion and >98% selectivity) with *tert*-butylhydroperoxide (TBHP) [68,69,70] as well as in the oxidation of substituted phenols (99% yield in trimethylbenzoquinone from trimethylphenol after 1 h) or in the epoxidation of cyclohexene (44% epoxide yield with >98% selectivity after 3 h) in the presence of hydrogen peroxide [71,72].

Similarly, Mayoral and co-workers reported the synthesis of Ti(IV)-silica catalysts by grafting Ti(OiPr)_4_. This catalyst showed promising results in the epoxidation of non-functionalized alkenes, dienes and allylic alcohols using *tert*-butylhydroperoxide as oxidant [73]. It is also an efficient catalyst for epoxidation with dilute aqueous hydrogen peroxide under mild conditions (23% epoxide yield after 1 h) [74,75] and with slow dropwise addition of oxidant [76]. In the latter case the contribution of direct epoxidation to the productive H_2_O_2_ consumption increases considerably with respect to one shot addition (from 21 to 56%), improving the selectivity.

Other kinds of transition metals may be grafted onto mesoporous silica using the metallocene route and active centers composed of isolated Mo(VI), Cr(VI) and VO(IV) have been described [77,78]. However, post-transition metal centres can be grafted as well. For instance, AlEt_2_Cl grafted on amorphous mesoporous silica showed excellent results in Diels-Alder reaction of carbonyl-containing dienophiles, due to the formation of strong Lewis acid sites [79,80]. In the reaction between methyl acrylate and cyclopentadiene, 90% of conversion and an *endo/exo* ratio of the products of 95:5 were obtained after 30 min. Again, isolated tin centres on MCM-41 were recently prepared by grafting SnMe_2_Cl_2_ via a post-synthesis treatment with subsequent calcination. The Sn single sites obtained in such way were compared to the ones obtained via direct synthesis. The sites, studied by DFT calculations and IR spectroscopy on probe molecules, proved to be active in the Baeyer–Villiger oxidation of adamantanone, with a TOF value of 160 h^-1^, very close to 165 h^-1^ obtained over Sn-BEA prepared as described above (Section 3.1) [81].

**Figure 7:**
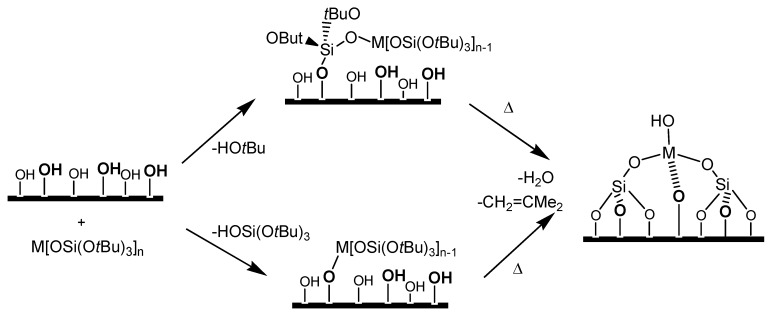
Preparation of single-site catalysts on mesoporous silica through thermolytic molecular precursors such as M[OSi(O*t*Bu)_3_]_n_. Adapted after Ref. [82].

Another complementary approach has been pioneered by Tilley and co-workers [82,83,84,85]. A molecular precursor was exploited to prepare a series of active catalysts on mesoporous supports (Figure 7) containing metal centres such as Ti, Cr, Fe or VO. In this case, the desired atomic environment aimed at in the final catalyst (e.g., Ti(OSi)_4_ or -Ti(OSi)_3_) was already present in the thermolytic precursor. Thus, for instance, starting from *tris*(*tert*-butoxy)siloxytitanium complex (iPrO)Ti-[OSi(OtBu)_3_]_3_, the local environment achieved at the end in the single-site catalyst is -Ti(OSi)_3_. Typical supports used by Tilley were the high-area mesoporous silicas MCM-41 and SBA-15, the latter being, for this purpose, distinctly more thermally stable than the former.

#### By varying the catalytically-active centre, it is possible to find applications for SSHC in a large choice of selective oxidations, following the guidelines of clean and sustainable chemistry. Figure 8 presents a range of single-site transition-metal ions grafted onto the surface of mesoporous silica [77,78,83,85,86].

**Figure 8:**
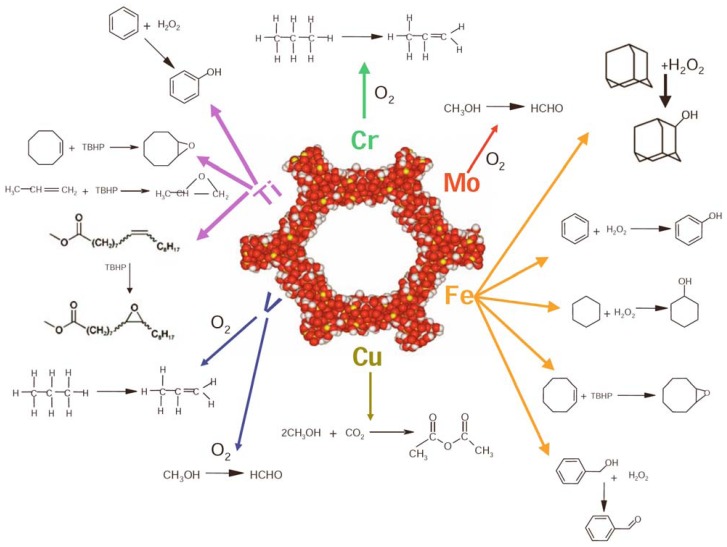
Some selective oxidations that can be carried out over SSHC obtained by depositing various metal centres onto the surface of hexagonal mesoporous silica. From Ref. [6], reproduced with kind permission of Springer Science and Business Media.

Another significant example of grafted single-site heterogeneous catalyst is the case of supported bimetallic catalysts [87]. They are formed by anchoring precursor metal-cluster carbonylates onto the inner walls of nanoporous silica supports. A gentle thermolysis converts the parent carbonylates to the ‘naked’ bimetallic nanoparticle catalysts. In this way it is possible to obtain individual species with a well-defined structure grafted in a spatially uniform manner in the inner walls of the supports.

The catalytic performance of bimetallic nanoparticles is generally far superior to that of the single metal alone. It was seen that supported Ru_6_Sn and Ru_6_Pd_6_ bimetallic nanoparticles are very active in the low-temperature selective hydrogenation of 1,5,9-cyclododecatriene, a key reaction for the industrial synthesis of valuable organic and polymeric intermediates [88]. Ru_6_Pd_6_ and Ru_6_Sn catalysts showed, under solvent-free conditions, TOF values of 5,350 h^-1^ and 1,940 h^-1^ respectively. Analogously, in the hydrogenation of 2,5-norbornadiene, the observed TOFs for both Ru_6_Sn and Ru_6_Pd_6_ were very large (10,210 h^-1^ and 11,176 h^-1^, respectively), with a norbornene to norbornane ratio of 8:1 over the former, but only of 1:3 over the latter. The bimetallic nanocatalyst Ru_10_Pt_2_ is particularly efficient in the single step hydrogenation of dimethylterephthalate (TOF value of 714 h^-1^) to give the desired 1,4-cyclohexane-dimethanol and dimethylhexahydroterephthalate [89]. 

### 3.3. Anchored organometallic complexes as SSHC

**Figure 9:**
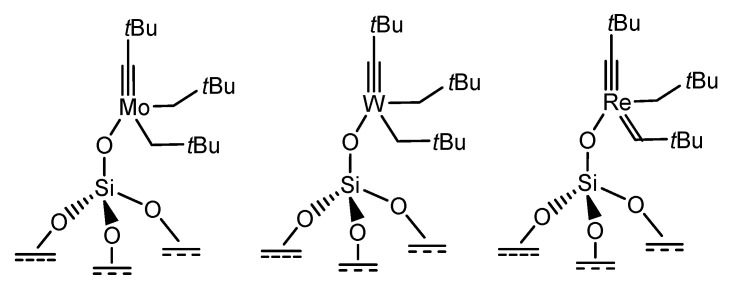
Single-site active centers designed to catalyse metathesis reactions and deposited onto a silica surface.

Different approaches have been introduced to immobilize on solid surfaces the active organometallic complexes which are designed and optimised to work under homogeneous conditions in liquid phase. Thanks to the *surface organometallic chemistry *approach, Basset and co-workers have produced a number of organometallic complexes anchored onto nonporous silica surfaces [4]. The organometallic complex is attached directly to the oxide support (silica, alumina, *etc.*) via either a covalent/ionic bond or a Lewis acid–Lewis base interaction. In these systems, the coordination sphere of the metal involves not only the ancillary ligands that influence the stability the activity and the selectivity like in homogeneous catalysis, but also the surface. Molybdenum, tungsten or rhenium carbyne complexes were, in this way, effectively anchored on the silica surface (Figure 9) and they are catalytically active in the metathesis of alkenes (with TOF value of 0.53 h^-1^ in the case of Mo catalyst) [90,94].

Another immobilization strategy is to link the transition metal complex catalyst to a mesoporous material via a spacer chain (a tether). This immobilization can be carried out by substituting one of the ligands of the homogeneous catalyst by a similar moiety, that contains a linker and that is able to anchor, typically via a covalent bond, to the support/carrier (oxide, polymer, dendrimer). According to this approach, most of the synthetic effort is devoted to preserve as much as possible the molecular structure and the chemical environment after anchoring [95].

**Figure 10:**
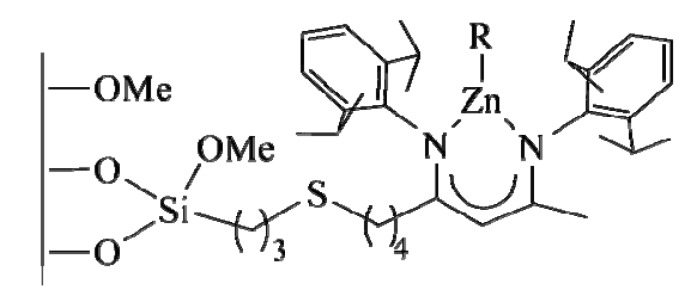
Zinc β-diiminate anchored onto mesoporous silica

For instance, in these years, Jones *et al.* have developed silica-tethered metal complex catalysts for polymerization reactions [96]. Zinc β-diiminate (Zn-BDI) complex was supported over two kind of silica support (SBA-15 and controlled pore glass, CPG) by following three synthetic strategies: 1) by anchoring the previously formed complex and tether onto the silica support, 2) by functionalising the silica support with the organic tether and the ligand and, then, by adding the active Zn site and 3) by functionalising the support with the tether alone and then by linking the metal + the ligand complex [97]. Such strategies led to the formation of the system in Figure 10, that is active in the copolymerization of carbon dioxide and cyclohexene oxide to produce poly(cyclohexene carbonate). All these catalysts effectively produced copolymer with varying amounts of homopolymer (polycyclohexene ether), as a side product. The catalyst formed by using the direct grafting of preformed complex showed the best result in terms of fraction of polycarbonate produced (65–78% of copolymer). One of the problems is the formation of undesired sites due to interactions between metal species and silanols of the surface.

In another example, the same team introduced a general strategy that led to well-defined aminosilica scaffold on the surface that is free from accessible silanols. Ti [98,99] and Zr [100] prepared according to the constrained geometry-inspired catalyst (CGC) approach, were immobilized on this scaffold that prevents metal-silanol interaction and leaves the metal centres fully exposed to the reactant. Such strategy improved the activity in the polymerization of polyethylene (PE) with respect to the homogeneous analogues. For silica-immobilized Ti CGCs the productivity was 28.7 kg PE (mol Ti h)^‑^^1^, compared to a value of 15.6 for the homogeneous systems [99].

**Figure 11:**
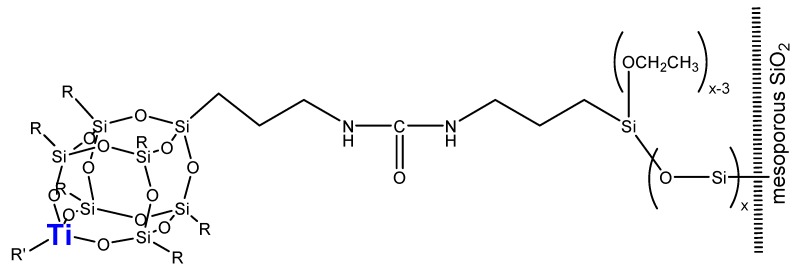
Anchoring of a Ti-containing polysilsesquioxane moiety onto the surface of SBA-15 silica (R = isobutyl; R’ = isopropyl group)

Thanks to the anchoring strategy, large and bulky moieties can be covalently attached onto the surface of mesoporous molecular sieves, provided the mesopores are large enough to accommodate the guest species. As an example, a Ti-containing polysilsequioxane (Ti-POSS), a complex widely used as a soluble model for Ti single sites in a silica matrix, was recently anchored via covalent bonding to the surface of a mesoporous SBA-15 silica (Figure 11). The final Ti-POSS-SBA-15 material revealed a good dispersion of the Ti sites and an interesting catalytic activity in the liquid-phase epoxidation of limonene to limonene oxide (with selectivity as high as 88% to 1,2-limonene oxide after 24 h) [101]. 

### 3.4. Single-site heterogeneous organometallic chiral catalysts

There is an ever-growing need for high-performance, heterogeneous, recyclable asymmetric catalysts. Nevertheless, none of the proposed systems so far has replaced the use of homogeneous catalyst at industrial scale. It is a desired objective to immobilize the homogeneous catalysts that are optimised for use under liquid phase conditions, in porous materials not only without losing their merits, but also enhancing and improving their stereoselective features. Actually, the catalytic performance of the solid chiral catalysts, particularly those accommodated within the pores or cavities of porous materials, are largely affected by the nature of the surface and the morphology of the porous network. In recent years, many new strategies have been proposed to accelerate the development of reliable, robust heterogeneous asymmetric catalysts, and many papers outline various possible strategies for ‘imprinting’ a surface with chirality [102,103,104,105,106,107,108,109,110,111,112].

A homogeneous chiral catalyst can be heterogenised on solid supports through various methods, such as grafting, ion-exchange or adsorption of a homogeneous catalyst on supports [5,105,106,113], encapsulation of chiral catalysts by ship-in-a-bottle method [114] or sol–gel method [115]. Among these methods, the immobilization of a chiral catalyst into the nanopores of inorganic supports is still an open question. A wide range of sizeable chiral metal complexes and organometallic moieties may be tethered to the inner walls of mesoporous silica either of the highly ordered (for example, MCM-41 and SBA-15) or non-ordered commercially available type.

**Figure 12:**
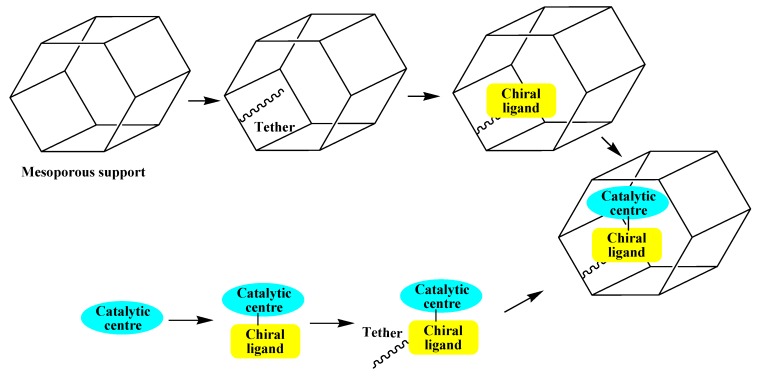
Two distinct approaches to anchor catalytically-active chiral metal complexes onto silica-based molecular sieves.

Generally, the post-synthesis modification is one of the most effective and largely used techniques [5,105,106]. For silica-based supports, the chiral metal complex can be introduced into the nanosized pores via a covalent or non-covalent linkage to the silanol groups on the silica surface. Two different anchoring methods are typically used: 1) *in situ* building (on the inner surface of the molecular sieve) of the asymmetric organic tether and, then, complexation of the catalytically-active metal centre or 2) *ex situ* assembling of the chiral metal complex and, then, anchoring of the pre-formed catalyst onto the inorganic support [5] (Figure 12).

In order to introduce the chiral catalysts exactly and only inside the nanopores, the silanol groups located on the external surface of the support can be previously ‘passivated’ (e.g. by selective silylation), so that the chiral catalysts are anchored exclusively via the reaction with the free silanol moieties on the internal surface [87].

Analogously, the non-covalent anchoring of asymmetric catalysts as suggested by Bianchini *et al*. [116] or de Rege *et al*. [117] is an effective method to generate well-defined, isolated, and readily accessible single sites for enantioselective conversions. The chiral *supported hydrogen-bonded* (SHB) rhodium catalysts, obtained by immobilization of metal chiral complexes on silica via hydrogen bonding, have been used so far in the enantioselective hydrogenation of prochiral olefins, particularly itaconates and 2-acetamido acrylates, in *n*-heptane or *n*-hexane [118]. As a general trend, the immobilization of the chiral precursors on silica did not reduce their enantioselectivity as compared to analogous homogeneous reactions in methanol and a beneficial effect was actually observed in some cases. Moreover, the conversions were generally comparable to or higher than those in homogeneous phase.

Irrespective of the immobilization procedure of the metal complex (SHB or ion pairing to SHB counter-anion), no appreciable rhodium leaching was observed within several consecutive heterogeneous runs, and an effective catalyst recycling with no loss of activity or enantioselectivity was accomplished by standard procedures.

**Figure 13:**
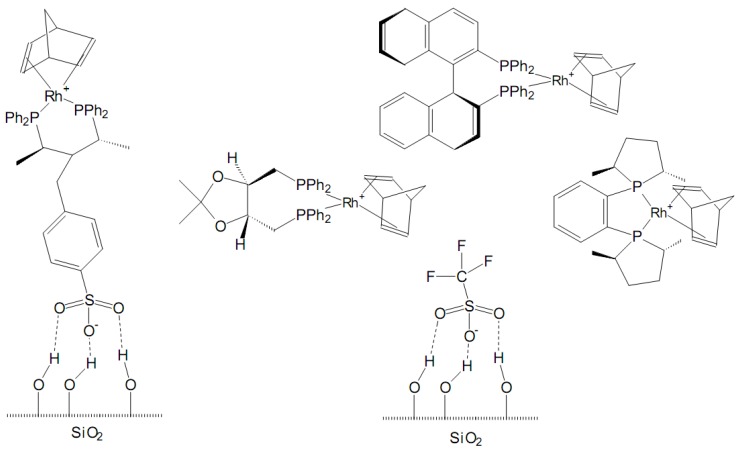
Neutral (zwitterionic) and ionic rhodium chiral complexes immobilized by hydrogen bonds over silica.

As mentioned above, the anchoring of an organometallic chiral catalysts may lead to a noteworthy impact on the catalytic behaviour. When certain kinds of chiral complexes are immobilised onto the inner walls of a mesoporous molecular sieve, it follows that the reactant’s (substrate) interaction with both the pore walls and the chiral directing group is distinct from the interaction it would experience if the chiral catalysts were free (as in the case of a purely homogeneous catalyst). This phenomenon has been ascribed to the *confinement effect*. In such scenario, the reactant within the pores is more largely influenced by the chiral directing groups in the proximity of the metal active centre, in terms of orientation of approach and asymmetric induction, than if it were in homogeneous solution without any steric constraint by the solid [87,119,120,121]. Essentially the confinement effect is a reflection of the interaction on the transition states leading to the chiral products. It may increase the enantioselectivity (positive effect) or decrease the enantioselectivity (negative effect) depending on how the interaction changes the transition states of the chiral products.

**Figure 14:**
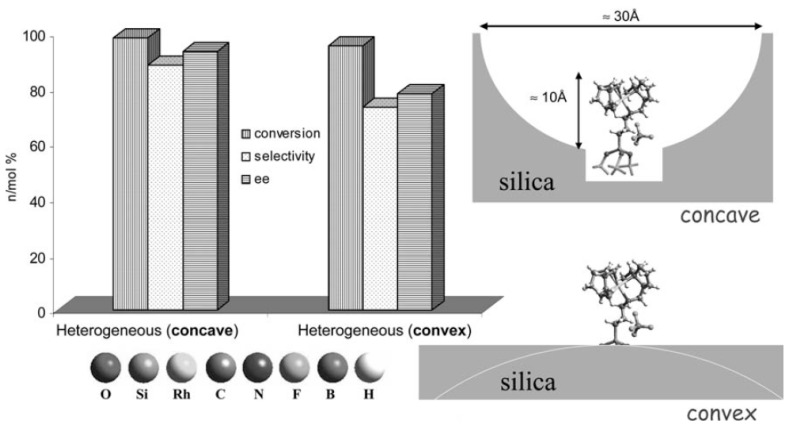
Sketch (to scale) of the influence of the confinement effect on the catalytic behaviour. The selectivity and *e.e.* value in the hydrogenation of *E*-(α)-phenylcinnamic acid obtained over the asymmetric Rh catalyst is higher in the spatially constrained system (concave surface) than in the less constrained one (convex surface). From Ref. [6], reproduced with kind permission of Springer Science and Business Media.

Figure 14 shows a remarkable example: tethering an asymmetric catalyst at a concave surface boosts the enantioselective behaviour [122]. When the hydrogenation of (*E*)-α-phenylcinnamic acid is performed over the same catalyst (Rh^I^-(*S*)-(-)-2-amino-methyl-1-ethyl pyrrolidine) tethered either on a concave or on a convex silica support, the differences in catalytic results, mainly in terms of enantioselectivity, are remarkable. Indeed the chiral induction, causing *e.e*. values higher than 95%, is maximised when the complex is anchored onto a concave silica surface and when there is thus a close interaction between substrate and chiral moiety as well as between substrate and pore walls. Analogously, Mn(salen) complexes, when immobilized inside siliceous nanopores, also exhibit the effect of enhancement of chirality owing to confinement effect in the enantioseletive epoxidation of unfunctionalized olefins [123].

## 4. Hybrid Catalysts, a Synergistic Combination of Supported Metal Nanoparticles and SSHCs

One of the major drawbacks shown by some SSHC, if compared with their homogeneous counterparts, is their lower activity. In most cases, such behaviour is due to mass transfer limitations that slow down the adsorption/desorption of reagents/products on active sites, thus lowering the activity of the catalysts. This problem becomes more and more important in three-phase processes, as hydrogenations, for instance, where a close contact of gas-phase hydrogen, liquid-phase reagents and solid-phase heterogeneous catalyst is required. A method to overcome such limitations, typically found in heterogeneous systems, while keeping their advantages can be found in the so called *hybrid catalysts*.

*Hybrid catalysts* consist of metal (nano)particles and metal coordination complexes adsorbed together on the same support material. They are a new class of heterogeneous catalysts able to combine, in a synergistic way, the advantages of classical heterogeneous (supported metal particles) systems with those of single-site systems (mostly grafted or anchored complexes). The co-presence of metal nanoparticles and molecular metal complexes develops a synergistic effect that increases the performance of such systems in several reactions. 

Hybrid catalysts based on metallic palladium adsorbed on a silica surface together with a rhodium complex were first reported in an early example by Barbosa *et al.* in 1977 [124] and successively explored by Gao and Angelici [125,126] and by Bianchini and Psaro since 1997 [127,128,129], for several organic reactions: e.g., reduction of ketones, hydrodehalogenation of fluorobenzene, hydroformylation of olefins and hydrogenation of arenes and olefins.

Several *tethered complexes on a supported metal* (TCSM) catalysts, based on Rh complexes and Pd NPs, were found very active in hydrogenation of arenes: Rh-dppp (dppp = (diphenyl-phosphino)propane) catalysts tethered via H-bonding on Pd/SiO_2_[130] showed TOF values up to 38.4 min^-1^ in the hydrogenation of benzene to cyclohexane.

Functionalized arenes, like methylbenzoate, phenol and anisole, can be efficiently hydrogenated with catalysts such as [Rh(COD)(N-N)]BF_4_/-Pd/SiO_2_ with bipyridyl ligands (N-N) and [Rh(COD)(N-P)]BF_4_/-Pd/SiO_2_ with pyridylphosphine ligands (N-P) [131,132]. Rh(N-N)-Pd/SiO_2_ catalysts was more active and versatile than Rh(N-P)-Pd/SiO_2_: TOF values up to 51 and 18.7 min^-1^ were obtained in the hydrogenation of anisole and methylbenzoate respectively. Rh(N-N)-Pd/SiO_2_ was more active towards the hydrogenation of the aromatic moiety and of the carbonyl group and Rh(N-P)-Pd/SiO_2_ more selective towards the hydrogenation of the carbonyl group. However, even such active systems show some drawbacks, as low reactivity with dimethylterephthalate and incomplete hydrogenation of acetophenone, the distribution of partially-hydrogenated products being strongly dependent on the catalyst and the reaction conditions.

The efficiency of the bimetallic catalyst system composed of a metal adsorbed on silica and a soluble rhodium complex tethered to the same support was further improved by entrapment of metallic palladium and rhodium precursor within a sol-gel matrix by Abu-Reziq [133]. Such systems, prepared by reduction of Na_2_PdCl_4_ with HSi(OEt)_3_ and addition of the palladium dispersion together with rhodium complex to a solution of Si(OEt)_4,_ were robust towards leaching, perfectly recyclable and did not require any hydrogen pretreatment.

In the catalytic hydrogenolysis of aromatic ketones good to excellent results were recorded for hydroxyacetophenone (88% conversion), acetophenone (79% conversion), benzophenone (69% conversion) and, in particular, for 1-(4-trifluoromethylphenyl)ethanol (95% conversion) [134]. 

The potential of hybrid catalysts was also exploited in enantioselective catalysis, in particular in asymmetric hydrogenation of prochiral olefins. The first examples of enantioselective catalysts, based on rhodium complexes containing the chiral ligand [(2*S*, 4*S*)-4-(diphenylphosphino)-2-(diphenyl-phosphinomethyl)pyrrolidine] (PPM) [135,136] in combination with Pd, were reported by Angelici for the asymmetric hydrogenation of the methyl-α-acetoamidocinnamate (MAC) [137]. Activities and enantioselectivities of the catalyst on Pd-SiO_2_ and on SiO_2_ support, were very similar in the hydrogenation of MAC (TOF = 11.8 min^-1^ and *e.e*. = 88.3% with Pd-SiO_2_, TOF= 11.2 min^-1^ and *e.e*. = 91% with SiO_2_, respectively).

Finally, in order to obtain a catalyst easy separable and reusable, a palladium catalyst has been encapsulated in silica sol-gel matrix modified with polyethylenimine together with ionic-liquid-modified magnetic nanoparticles [138]. A complete conversion obtained in the one-pot hydrogenation of benzyl alcohols and the possibility to reuse the palladium-based catalyst, after magnetic separation, to catalyse different reactions, such as carbonylation of iodoarenes (with conversions in the range from 97 to 100%), Suzuki coupling (88 to 100% conversion) and Heck reaction between 4-iodo-acetophenone and *n*-butylacrylate (95% conversion) without any loss of activity, made this catalyst an attractive system.

## 5. Conclusions

The examples presented in this brief overview show how SSHC can be applied to different reactions in fine chemicals synthesis. Since the fundamental discovery of TS-1 zeolite and the development of metal-containing aluminophosphates and silicoaluminophosphates (Me-ALPO and Me-SAPO), redox-active metal-containing solid catalysts can be used as viable alternatives to stoichiometric non-catalytic processes (still widely used, especially in oxidation reactions) that rely on hazardous and poorly sustainable reactants. Such kind of catalysts, obtained via direct synthesis, proved to be robust and easily reusable and they are, in general, the preferred candidate for developing new synthetic processes at large-scale level.

On the other hand, from the point of view of the organic chemist, the post-synthesis modification of a previously prepared support material or, in the luckiest cases, of a commercial inorganic solid can be a powerful tool to obtain relatively quickly a series of heterogeneous catalysts with tailored and controlled features (e.g., by varying nature, content or distribution of the catalytically active metals) for a rapid screening in chemo-, regio- and stereoselective transformations. However, at present, several research papers do not pay enough attention to critical issues, such as robustness to deactivation, easy preparation and availability of the SSHC materials and this is a major drawback for an effective boost of the use of SSHC systems in industrial production.

So, the development of chemically stable, easily recyclable and selective single-site heterogeneous catalysts based on nanostructured molecular sieves is still a challenge for researchers in many domains of selective organic synthesis. However, the tremendous potential of SSHCs in terms of achievable activity and selectivity under mild and ‘green’ conditions should be stressed even more. The recent set up of production plants, at an industrial level, of mesoporous nanostructured silica materials could path the way to a wider exploitation of SSHCs for the transformation of fine chemicals too. In this aim, a multidisciplinary approach, based on a strict cooperation between chemists with different cultural backgrounds (in organic synthesis, inorganic chemistry, materials sciences, *etc*.) will be the key to obtain successful results. 
